# Multicentre evaluation of the Monarch (IL) clinical chemistry analyser

**DOI:** 10.1155/S1463924689000416

**Published:** 1989

**Authors:** Ferruccio Ceriotti, Emma Guagnellini, Francesco Zoppi, Norberto Montalbetti, Carlo Okely, Pierangelo Bonini

**Affiliations:** 1 Istituto Scientifico H. S. Raffaele Via Olgettina 60 Milano 20132 Italy; 2 Ospedale Bassini Cinisello Balsamo Milano 20132 Italy; 3 Ospedale Niguarda Ca' Granda Milano 20132 Italy

## Abstract

A multicentre evaluation of the Monarch centrifugal analyser is reported. Precision, linearity and accuracy were assessed by comparison with routine methods. Calibration stability, photometric and dispensing accuracy, and carry-over related to samples and reagents were also evaluated. The overall performance of the instrument was good, showing an excellent photometric and dispensing accuracy, absence of sample-dependent carry-over, and almost negligible reagent carry-over. Good precision, linearity and correlation with routine methods were found for the parameters tested. The instrument is reliable and is now used as the routine clinical chemistry analyser in two of the three laboratories taking part in the evaluation.


Journal of Automatic Chemistry Vol. 11, No. 5 (September–October 1989), pp. 206–211

### Multicentre evaluation of the Monarch (IL) clinical chemistry analyser

**Ferruccio Ceriotti, Emma Guagnellini†, Francesco Zoppi‡, Norberto Montalbetti‡, Carlo Okely† and Pierangelo Bonini**

*Istituto Scientifico H. S. Raffaele, Via Olgettina 60, 20132 Milano (Italy)*
*† Ospedale Bassini, Cinisello Balsamo ‡ Ospedale Niguarda Ca' Granda*

*A multicentre evaluation of the Monarch centrifugal analyser is reported. Precision, linearity and accuracy were assessed by comparison with routine methods. Calibration stability, photometric and dispensing accuracy, and carry-over related to samples and reagents were also evaluated. The overall performance of the instrument was good, showing an excellent photometric and dispensing accuracy, absence of sample-dependent carry-over, and almost negligible reagent carry-over. Good precision, linearity and correlation with routine methods were found for the parameters tested. The instrument is reliable and is now used as the routine clinical chemistry analyser in two of the three laboratories taking part in the evaluation.*

*Keywords: Instrument evaluation; centrifugal analyser.*

### Introduction

The Monarch is an automatic random access centrifugal analyser for clinical chemistry determinations. Random analysis of samples is possible using a robotic system to

*Table 1. Methods used on the Monarch and on the comparison instrument.*

| | Monarch | | | | | Comparison methods | | |
|---|---|---|---|---|---|---|---|---|
| | | | Volumes (μl) | | | | | |
| Parameters tested | Principles | Wavelength (nm) | Sample | Reagent(s) | Company | Principles | Company | Analyser |
| Glucose | hexokinase/G6PDH (EP) | 340/380 | 3 | 200 | IL | hexokinase/ G6PDH | BM† | Hitachi 737 |
| Cholesterol | chol. oxid./trinder (EP) | 500/690 | 2 | 200 | IL | chol. oxid./trinder | Miles | Hitachi 737 |
| Total bilirubin | sulphanilic ac. SDS (EP) | 550/620 | 8 | 50/72 | IL (Italy) | Jendrassik | BM | Hitachi 737 |
| Urate | uricase/trinder (EP) | 550/690 | 4 | 100 | IL (Italy) | – | | |
| Total protein | biuret (EP) | 550/620 | 5 | 200 | IL | – | | |
| Calcium | cresolphtalein complexone (EP) | 570/650 | 4 | 180 | IL | – | | |
| Phosphate | unreduced phosmolibdate (EP) | 340/520 | 4 | 150 | IL (Italy) | – | | |
| Triglyceride | lipase/glycerol kinase/UV (FX) | 340 | 3 | 150 | IL | lipase/glycerol trinder | Miles | Hitachi 737 |
| Creatinine | picric acid (FX) | 520 | 9 | 150 | IL | picric acid kinetic | BM | Hitachi 737 |
| Urea | urease/GLDH (FX) | 340 | 2 | 200 | IL | urease/GLDH | BM | Hitachi 737 |
| AST | Scandinavian Soc. 37 °C (K) | 340 | 16 | 100/37 | IL (Italy) | IFCC 37 °C without P5P | BM | Hitachi 737 |
| ALT | Scandinavian Soc. 37 °C (K) | 340 | 16 | 100/37 | IL (Italy) | IFCC 37 °C without P5P | BM | Hitachi 737 |
| ALP | Scandinavian Soc. 37 °C (K) | 405 | 2 | 150/50 | IL (Italy) | diethanolamine buffer 37 °C | BM | Hitachi 737 |
| Sodium | I.S.E. | – | 30 | 1080 | IL | flame photometry | IL | IL 943 |
| Potassium | I.S.E. | – | 30 | 1080 | IL | flame photometry | IL | IL 943 |
| Chloride | I.S.E. | – | 30 | 1080 | IL | coulometry | Eppendorf | 6610 |
| Magnesium | calmagite (EP) | 650/570 | 2 | 200 | Lancer | atomic absorption | Pie Unicam | SP9000 |
| Iron | ferene-S (EP) | 600/690 | 25 | 60/60 | Sentinel | ferrozine | Merck | ERIS |

*Notes:* EP = end-point; FX = fixed time; and K = kinetic. † Boehringer Mannheim.

0142–0453/89 $3.00 © 1989 Taylor & Francis Ltd.

change the disposable cuvette rotors automatically. The disposable reaction rotors, made of UV-transmitting plastic material, contain 39 usable optical cuvettes, all with a pathlength of 0·744 ± 0·013 cm (and a reference cuvette). The instrument can deliver sample volumes between 2 and 89 μl with a minimum reaction volume of 150 μl and a maximum of 260 μl. It is able to perform colorimetric, nephelometric and fluorimetric analyses, and, in a separate module of the instrument, potentiometric tests. The optical unit consists of a monochromator (wavelength between 300 and 800 nm), and 12 interference filters (from 340 to 690 nm) with a bandwidth of about ±1·5 nm. An optional ISE module determines sodium, potassium and chloride using the indirect potentiometric technique.

The Monarch has a refrigerated reagent tray (15 °C) that can hold up to 20 wedge-shaped reagent boats (maximum capacity 16 ml); the sample ring holds up to 44 cups, 38 sample or control positions and six calibrator positions. The instrument is programmed via a keyboard and a video display unit with a user-friendly menu.

The theoretical throughput of the instrument is about 400 results/h when performing photometric analyses, and about 600 results/h when the ISE module is included. The Monarch automatically organizes the analytical cycle to maximize throughput, which depends upon the number of samples, the number of analyses per sample and whether one or two reagents are being used.

The evaluation reported here took photometric analyses (biochromatic equilibrium, fixed time, kinetic) and the ISE module into account. Additionally, general features, such as photometric performance and dispensing and diluting accuracy using a bichromate solution, were considered.

The work was done on three different instruments installed in three laboratories. For practical and organizational reasons it was not possible to perform every experiment in each laboratory, as defined in the ECCLS multicentre evaluation protocol [1]; therefore each participating laboratory performed a different part of the evaluation, but some critical tests were repeated in more than one laboratory.

### Materials and methods

*Reagents*

Table 1 shows the reagent and methods used both on Monarch and the comparison instrument.

The Monarch was calibrated with four different calibrators:

(1) ReferrIL A (lot No. 6071) for glucose, urea, creatinine, phosphate, sodium, potassium and chloride (lab 1).

(2) ReferrIL B (lot No. 6051) for cholesterol, total protein, sodium, potassium and chloride (lab 1).

(3) ReferrIL 3 C (lot No. 6071) for bilirubin (lab 1) and urate.

(4) Ultimate (Beckman lot No. M 791935) for bilirubin (lab 2).

The within- and between-batch imprecision was evaluated using the following materials:

(1) Pool of normal sera, subdivided in aliquots and stored at −20 °C.

(2) Precinorm U lot No. 153148 (Boehringer Mannheim).

(3) Precipath U lot No. 152794 (Boehringer Mannheim).

(4) Serachem Lipid lot No. 221085 (Fisher Scientific Orangeburg, New York 10962, USA) (only for cholesterol and triglycerides – high level).

*Table 2. Imprecision of the Monarch.*

| | *Level I* | | | | *Level II* | | | | *Level III* | | | |
|---|---|---|---|---|---|---|---|---|---|---|---|---|
| | Mean | CVw (%) | CVb (%) | CVo (%) | Mean | CVw (%) | CVb (%) | CVo (%) | Mean | CVw (%) | CVb (%) | CVo (%) |
| Glucose (mmol/l) | 5·026 | 1·73 | 1·47 | 2·27 | 6·602 | 1·60 | 1·29 | 2·06 | 14·848 | 0·94 | 1·02 | 1·38 |
| Cholesterol (mmol/l) | 2·886 | 3·21 | 4·09 | 5·20 | 5·191 | 2·21 | 2·96 | 3·70 | 8·023 | 1·53 | 2·20 | 2·68 |
| Total bilirubin (μmol/l) | 10·76 | 1·85 | 2·28 | 2·93 | 38·39 | 1·18 | 2·03 | 2·35 | 98·15 | 0·59 | 2·41 | 2·48 |
| Triglyceride (mmol/l) | 0·982 | 1·95 | 2·50 | 3·17 | 1·149 | 2·42 | 2·36 | 3·38 | 3·392 | 1·37 | 1·95 | 2·38 |
| Urea (mmol/l) | 6·173 | 2·01 | 3·99 | 4·47 | 10·106 | 2·39 | 2·44 | 3·42 | 22·67 | 1·40 | 2·19 | 2·60 |
| Creatinine (μmol/l) | 86·45 | 2·84 | 4·29 | 5·20 | 183·87 | 2·03 | 2·61 | 3·30 | 296·67 | 1·48 | 1·83 | 2·35 |
| AST (U/l) | 17·50 | 2·86 | 2·13 | 3·57 | 59·00 | 1·35 | 2·08 | 2·48 | 130·40 | 0·96 | 1·66 | 1·92 |
| ALT (U/l) | 11·87 | 4·35 | 4·89 | 6·55 | 48·00 | 1·35 | 0·39 | 1·41 | 112·42 | 0·75 | 0·95 | 1·21 |
| ALP (U/l) | 174·91 | 1·28 | 1·18 | 1·74 | 333·93 | 2·80 | 2·53 | 3·77 | 529·40 | 1·74 | 1·34 | 2·20 |
| Na (mmol/l) | 119·43 | 0·47 | 0·74 | 0·87 | 136·69 | 0·52 | 0·70 | 0·88 | 143·14 | 0·51 | 0·69 | 0·86 |
| K (mmol/l) | 4·308 | 0·61 | 1·01 | 1·18 | 4·437 | 0·47 | 0·82 | 0·94 | 6·320 | 0·54 | 1·12 | 1·25 |
| Cl (mmol/l) | 101·08 | 0·50 | 0·88 | 1·01 | 106·52 | 0·44 | 0·90 | 1·01 | 121·40 | 0·51 | 1·08 | 1·19 |

*Notes:* w, within batch; b, between batch; o, overall.

*Table 3. Calibration stability.*

| | Period No. days | No. results | Slope | *r* | Trend probability *p*† |
|---|---|---|---|---|---|
| Glucose | 68 | 37 | −0·425 | −0·0699 | 0·677 |
| Urea | 69 | 38 | 3·093 | 0·4308 | 0·007‡ |
| Creatinine | 57 | 30 | 0·251 | 0·3265 | 0·078 |
| Cholesterol | 67 | 41 | −0·928 | −0·1303 | 0·417 |
| Urate | 71 | 38 | 0·107 | 0·0852 | 0·610 |
| Total bilirubin | 54 | 36 | 0·173 | 0·1688 | 0·320 |
| Total protein | 71 | 41 | 0·120 | 0·1020 | 0·526 |
| Calcium | 69 | 36 | 4·221 | 0·3913 | 0·018‡ |
| Phosphate | 64 | 40 | 2·268 | 0·2994 | 0·060 |
| Magnesium | 36 | 32 | −0·397 | −0·4435 | 0·011‡ |
| Iron | 30 | 25 | 0·052 | 0·3178 | 0·121 |

† Probability that the slope significantly differs from 0, calculated by Student's *t*-test.
‡ Significant trend probability.

*Table 4. Photometric accuracy; bichromate solution analysed with the pre-load mode.*

| Dilution | Theoretical values Abs† | Instrument 1 Abs | (CV%) | Δ% | Instrument 2 Abs | (CV%) | Δ % |
|---|---|---|---|---|---|---|---|
| 1 | 0·667 | 0·690 | (0·4) | +3·45 | — | | |
| 2 | 0·995 | 1·010 | (0·6) | +1·51 | — | | |
| 3 | 1·320 | 1·310 | (0·5) | −0·76 | — | | |
| 4 | 1·6413 | 1·650 | (0·5) | +0·53 | 1·620 | (0·3) | −1·3 |

† Obtained on Uvikon 860 spectrophotometer.

Quality control materials were reconstituted at the beginning of each working day.

*Bichromate stock solution:* 20 mmol/l of potassium bichromate in $H_2SO_4$ 0·01 N.

### *Experimental design*

*Imprecision:* materials at three different levels of concentration were analysed five times per day for 10 days.

*Calibration stability:* the absorbances of the calibrators were recorded during a period of 30 to 71 days, and a regression analysis was performed to evaluate the possible presence of any significant trends.

*Photometric and dispensing accuracy:* the rotor was loaded manually, independently of the instrument, with several dilutions of the bichromate stock solution and then analysed with the 'pre-load' mode (this option enables the instrument to be used just as a photometer) using a wavelength of 340/690 nm. The theoretical absorbance of the bichromate solutions were obtained on an Uvikon 860 spectrophotometer (Kontron Instruments). The same solution as above, and a dilution of 1 : 5 of it, were assayed as samples using increasing sample volumes (from 2 to 20 μl), diluted with a constant volume of 180 μl of distilled water; a whole rotor was run for each tested volume. Ten to 100 μl of a 1 : 37 dilution of the same solution were then dispensed with the reagent pipette (setting the sample volume to zero) and diluted with a constant volume of 100 μl of distilled water.

*Table 5. Sample dispensing accuracy: bichromate solution analysed as sample.*

| Dilution | Reference values Abs† | Instrument 1 Abs | (CV%) | Δ% | Instrument 2 Abs | (CV%) | Δ % |
|---|---|---|---|---|---|---|---|
| 2 | 0·690† | 0·680 | (1·8) | −1·47 | 0·710 | (0·7) | +2·90 |
| 3 | 1·010† | 1·010 | (1·4) | 0·00 | 1·010 | (0·4) | 0·00 |
| 4 | 1·310† | 1·310 | (1·0) | 0·00 | 1·310 | (0·6) | 0·00 |
| 5 | 1·650† | 1·630 | (0·9) | −1·21 | 1·620 | (0·6) | −1·82 |
| 5‡ | 0·3273 | 0·320 | (2·2) | −2·20 | 0·303 | (1·5) | −7·42 |
| 10‡ | 0·6395 | 0·630 | (0·8) | −1·49 | 0·624 | (1·1) | −2·42 |
| 15‡ | 0·9341 | 0·920 | (0·7) | −1·51 | 0·927 | (0·9) | −0·76 |
| 20‡ | 1·2110 | 1·210 | (0·8) | −0·08 | 1·214 | (0·9) | +0·25 |

† Obtained on the Monarch n.1 with the 'pre-load' mode (see text).
‡ Bichromate solution diluted 1 : 5, theoretical absorbances calculated from the Uvikon values.

*Table 6. Dispensing accuracy: bichromate as reagent.*

| µl | Theoretical values Abs | Instrument 1 Abs | (CV%) | Δ% | Instrument 2 Abs | (CV%) | Δ % |
|---|---|---|---|---|---|---|---|
| 10 | 0·1492 | 0·150 | (1·2) | +0·54 | 0·143 | (4·2) | −4·16 |
| 20 | 0·2735 | 0·260 | (0·8) | −4·92 | 0·265 | (4·2) | −3·11 |
| 30 | 0·3793 | 0·360 | (0·4) | −5·09 | 0·362 | (1·3) | −4·56 |
| 50 | 0·5471 | 0·525 | (0·5) | −4·04 | 0·512 | (0·5) | −6·42 |
| 80 | 0·7290 | 0·710 | (0·3) | −2·61 | 0·686 | (0·6) | −5·90 |
| 100 | 0·8200 | 0·790 | (0·4) | −3·66 | 0·770 | (0·6) | −6·10 |

*Table 7. Method linearity.*

| Analyte | Range tested | Claimed limits | $r^2$ | Curvilinearity probability† |
|---|---|---|---|---|
| Glucose | 4·4–38·8 mmol/l | 27·7 | 0·99953 | 0·1221 |
| Triglyceride | 0·9–12·2 mmol/l | 11·3 | 0·99897 | 0·0346 |
| Total bilirubin | 5·1–431 µmol/l | 340 | 0·99995 | 0·1346 |
| Urea | 4·8–30·8 mmol/l | 33·3 | 0·99963 | 0·0868 |
| Creatinine | 97·2–1485 µmol/l | 1326 | 0·99204 | 0·3504 |
| AST | 23–191 U/l | 300 | 0·99979 | 0·0825 |
| ALT | 27–393 U/l | 300 | 0·99990 | 0·0500 |
| ALP | 123–1350 U/l | 1000 | 0·99916 | 0·1941 |
| Sodium | 99–158 mmol/l | 120–160 | 0·99981 | 0·6810 |
| Potassium | 1·6–15·2 mmol/l | 2·0–8·0 | 0·99900 | 0·8000 |
| Chloride | 80–173 mmol/l | 75–120 | 0·99900 | 0·1000 |

† According to Burnett and Martin [3, 4].

*Method linearity:* samples containing high levels of analyte were diluted, in varying proportions, with sera with low levels of analyte. Each dilution was then measured in duplicate.

*Method comparison:* 60–120 patient samples were analysed on the Monarch, in five–10 runs, over three weeks for each analyte. Results were compared with those obtained by methods and instruments in routine use in the evaluators' laboratories (see tables 1 and 8).

*Specimen-dependent carry-over:* this was assessed by running six sequences of three high samples (H) followed by three low ones (L). The carry-over was calculated using the formula:

$$\frac{L1 - (L2 + L3)/2}{H3 - (L2 + L3)/2}$$

*Method-to-method carry-over:* this was evaluated by analysing a mid-level human serum pool, in triplicate, so that

*Table 8. Method comparison.*

| Analyte | | N | $\bar{x}$ | $\bar{y}$ | Slope | Intercept | r | Sxy |
|---|---|---|---|---|---|---|---|---|
| Glucose | mmol/l | 116 | 6·199 | 6·066 | 0·9782 | 0·014 | 0·9965 | 0·2342 |
| Cholesterol | mmol/l | 129 | 4·431 | 4·377 | 1·0000 | −0·078 | 0·9876 | 0·2125 |
| Total bilirubin | µmol/l | 60 | 60·14 | 59·05 | 0·9670 | −0·735 | 0·9988 | 5·2670 |
| Triglyceride | mmol/l | 129 | 1·678 | 1·485 | 0·9024 | −0·046 | 0·9952 | 0·1110 |
| Urea | mmol/l | 124 | 7·349 | 7·680 | 1·0263 | 0·138 | 0·9938 | 0·3940 |
| Creatinine | µmol/l | 125 | 99·0 | 106·1 | 1·0796 | −0·619 | 0·9933 | 4·9504 |
| AST | U/l | 122 | 34·65 | 33·34 | 0·8727 | 3·06 | 0·9991 | 1·5482 |
| ALT | U/l | 123 | 36·63 | 39·33 | 1·0156 | 2·66 | 0·9987 | 1·9926 |
| ALP | U/l | 120 | 278·5 | 245·1 | 0·8258 | 14·59 | 0·9996 | 6·1573 |
| Sodium | mmol/l | 84 | 141·7 | 140·1 | 0·9375 | 7·20 | 0·9346 | 1·1942 |
| Potassium | mmol/l | 84 | 4·34 | 4·21 | 0·9732 | −0·006 | 0·9970 | 0·0443 |
| Chloride | mmol/l | 115 | 104·3 | 104·2 | 1·0000 | 0·000 | 0·9730 | 1·2314 |
| Magnesium | mmol/l | 80 | 0·85 | 0·885 | 0·9800 | 0·045 | 0·9790 | 0·0505 |
| Iron | µmol/l | 150 | 13·80 | 13·87 | 0·9870 | 0·234 | 0·9880 | 1·2351 |

each chemistry was preceded and followed by the other. It was considered that no carry-over had occurred if the variations in a certain sequence were within twice the CV of the method obtained in the within-batch precision.

### Results and discussions

### *Imprecision*

The different components of the imprecision were calculated according to the analysis of variance [2]; the results are shown in table 2. The within-run precision was acceptable in the majority of cases. The overall precision of the electrolyte determinations was excellent (CVs always lower than 1% in the case of sodium, and lower than 1·25 for potassium and chloride) and was good for every other analyte tested, with the exception of cholesterol and urea at low concentrations.

### *Calibration stability*

Results are shown in table 3. The stability of the calibration seemed to be good, and a significant trend exists only in the case of urea, calcium and magnesium. For these analytes a daily calibration is advised.

### *Photometric and dispensing accuracy*

As shown in table 4, the accuracy of the photometric system seemed acceptable. Also when bichromate was dispensed as a sample, the results were good (table 5); however, when it was used as a reagent, both instruments showed a consistent negative bias (table 6). This is not very important if the instrument is calibrated with external calibrators. When using the diluted bichromate solution (see table 5 – second half, and table 6), the experimental design does not allow the operator to distinguish between imprecision and inaccuracy due to the photometric system or to the dispensing device. The absorbances obtained were therefore compared with the theoretical value for a similar dilution of the solution. So it is possible to evaluate the overall accuracy of the system, but the components of any imprecision cannot be identified. The linearity of response was also calculated using the formulas proposed by Burnett and Martin [3, 4]. In each of the three cases, the curvilinear probability was not significant (pre-load mode $p = 0{\cdot}5110$ $r^2 = 0{\cdot}9996$, bichromate as sample $p = 0{\cdot}11418$ $r^2 = 0{\cdot}9996$, bichromate as reagent $p = 0{\cdot}3371$ $r^2 = 0{\cdot}9998$.

### *Linearity*

As shown in table 7, the linearity of the IL methods was very good and almost always higher than that claimed by the manufacturer.

### *Carry-over*

The specimen-dependent carry-over was found to be negligible for all the methods studied (table 9). A significant method-to-method carry-over was found only when total protein determination was followed by urate measurement. In this case, the urate value was reduced.

*Table 9. Sample-dependent carry-over.*

| Analyte | High pool | Low pool | % carry-over |
|---|---|---|---|
| Glucose | 64·93 mmol/l | 1·28 mmol/l | 0·03 |
| Cholesterol | 6·73 mmol/l | 1·66 mmol/l | 0·30 |
| Total bilirubin | 241 µmol/l | 6·5 µmol/l | 0·07 |
| Triglyceride | 8·25 mmol/l | 0·95 mmol/l | 0·06 |
| Urea | 40·0 mmol/l | 3·83 mmol/l | 0·05 |
| Creatinine | 1200 µmol/l | 79·6 µmol/l | 0·24 |
| AST | 269 U/l | 18 U/l | 0·02 |
| ALT | 381 U/l | 23 U/l | 0·10 |
| ALP | 845 U/l | 69 U/l | 0·01 |
| Sodium | 162 mmol/l | 110 mmol/l | 0·04 |
| Potassium | 5·0 mmol/l | 3·8 mmol/l | 0·60 |
| Chloride | 120 mmol/l | 84 mmol/l | 0·00 |

This is caused by a falsely elevated reagent blank. Should such a combination occur during calibration, an important increase of all the urate values would be observed. The absorbance increase of an urate reagent blank after a total protein assay (0·046 Abs) was similar to that found by adding one part of biuret reagent to 500 parts of urate reagent and reading the absorbance after an incubation of 5 min at 37 °C (0·052 Abs). Therefore, a reagent carry-over of about 1/500 can be assumed; this is evident only when particular reagent combinations occur. No carry-over was found when an ALT was followed by an LDH assay (a combination that is highly critical in other random access analysers [5]), nor was there any carry-over with any other combination of the analytes tested.

### *Method comparison*

Data and correlation parameters are presented in table 3. The regression line was computed with the non-parametric linear regression model of Passing and Bablock [6]. The correlation coefficient ($r$) was good, or very good, in every case (only for sodium was it somewhat lower, but the range tested was very narrow). The slope of the regression line shows that, in the cases of triglyceride, AST and ALP, there was an important negative proportional bias. This bias could be explained for triglyceride because of the differnt kinds of method and calibration material used and for AST and ALP by the different optimization of the methods, and the use (by the Hitachi 737) of a calibrator for enzymatic analyses.

### Conclusions

The overall performance of the apparatus seemed to be satisfactory. The instrument is extremely flexible and suitable for development and application of new reagents and research methods, and, moreover, showed a good reliability. It is now used as the routine clinical chemistry analyser in two of the three laboratories who took part in the evaluation.

### Acknowledgements

The authors wish to thank Dr P. Iandolo and Dr G. Luise (IL Company) for their help and fruitful discussion. The research reported here was carried out with the support of CNR (National Council for Research).

### References

1. *Guidelines for the Evaluation of Analysers in Clinical Chemistry* (ECCLS Document, Volume 3, Number 2, June 1986).
2. Krouwer, J. S. and rabinowitz, R., *Clinical Chemistry,* **30** (1984), 290.
3. Burnett, R. W., *Clinical Chemistry,* **26** (1980), 644.
4. Martin, R. F., *Clinical Chemistry,* **26** (1980), 1509.
5. Degenaar, C. P., *Clinical Chemistry,* **29** (1983), 1649
6. Passing, H. and Bablock, W., *Journal of Clinical Chemistry and Clinical Biochemistry,* **21** (1983), 709.

### PROCESS ANALYSIS: A ROAD TO SAFETY AND EFFICIENCY

*A residential school to be held from 29 April to 4 May 1990 at University of Warwick, UK*

The Institute of Measurement and Control is running the twelfth in a successful series of schools on process analytical instrumentation. It aims to provide participants with an updated knowledge of current analytical techniques and an understanding of how automated chemical analysis aids profitability, safety and environmental control through a number of lectures and syndicate project work.

The schools serve a useful purpose and meet a real demand by a number of industries such as the chemical, petrochemical, metal manufacturing, power generation, food processing and process industries using analytical instruments to profitable and safe effect.

The 1990 fees which include attendance, accommodation, meals, and documentation for the duration of the course will be £650 for members and £700 for non-members plus VAT.

Lecture topics will include: The Case for Process Analysis; System Design Considerations; Sampling; Signal Processing, Data Handling and Communications; Calibration and Validation; Cost Effective Maintenance of Analysers; Electroanalytical Principles and Applications; Spectrophotometric Analysis; Chromatography; Mass Spectrometry; Efficient Combustion and Pollution Control by Gas Analysis; Measurement of Water and Effluent Streams; Monitoring Gaseous Effluents; Flammable Gas Detectors; Chemometrics; Safety Considerations; Miscellaneous Analytical Techniques; and A Review of Future Trends in Process Analytical Instrumentation.

*Further details can be obtained from: The InstMC, 87 Gower Street, London WC1E 6AA, UK. Tel.: (01) 387 4949.*

